# Tuina combined with other therapies for treating insomnia: a systematic review and network meta-analysis

**DOI:** 10.3389/fneur.2026.1855450

**Published:** 2026-05-28

**Authors:** Zihang Tong, Shun Fan, Yusheng Li, Qiaoling Chen, Huanan Li, Jingui Wang

**Affiliations:** 1 First Teaching Hospital of Tianjin University of Traditional Chinese Medicine, Tianjin, China; 2 National Clinical Research Center for Chinese Medicine, Tianjin, China; 3 Level 3 Laboratory of Biological Effects of Tuina Manipulation of National Administration of Traditional Chinese Medicine, Tianjin, China; 4 Tianjin Institute of TCM Tuina of Tianjin Municipal Health Commission, Tianjin, China

**Keywords:** insomnia, massage, network meta-analysis, sleep, tuina

## Abstract

**Background:**

Insomnia has a high incidence rate among adults, severely affecting physical and mental health and increasing the risk of multiple diseases. Tuina represents an effective non-pharmacological intervention for insomnia management. Nevertheless, the majority of existing clinical evidence on Tuina for insomnia pertains to its use in conjunction with other modalities, and the relative effectiveness among different combined approaches remains unknown. Accordingly, this network meta-analysis compared the impacts of various Tuina-based combination therapies on patients with confirmed insomnia.

**Methods:**

A systematic search was performed across PubMed, Cochrane, Embase, Web of Science, CNKI, VIP, WanFang, and SinoMed electronic databases to identify randomized controlled trials (RCTs) meeting the prespecified criteria. Network meta-analysis was performed using Stata version 16.0.

**Results:**

Thirty-four RCTs comprising 2,663 subjects were enrolled, assessing 12 distinct Tuina-based combined interventions. The network meta-analysis revealed that for the outcome of total effective rate, the following combinations were showed a statistically significant difference compared to oral medication (*P* < 0.05): Tuina+breath guiding+acupoint application, Tuina+music, Tuina+foot bath, Tuina+breath guiding, Tuina+foot bath+acupoint application, Tuina+acupuncture, Tuina+acupoint application, and Tuina+scraping. The top-ranked regimens were Tuina+breath guiding+acupoint application, Tuina+music, and Tuina+foot bath. For the outcome of Pittsburgh Sleep Quality Index (PSQI) score improvement, Tuina+breath guiding+acupoint application, Tuina+music, Tuina+foot bath, and Tuina+acupuncture were significantly better than oral medication (*P* < 0.05). The three highest-ranking interventions were again Tuina+breath guiding+acupoint application, Tuina+music, and Tuina+foot bath. Regarding safety, Tuina+acupoint application exhibited the lowest rate of adverse events; however, this finding was derived from only 5 RCTs assessing 3 interventions, and the sparse evidence network precludes definitive comparative safety conclusions.

**Conclusion:**

Tuina-based combination therapies showed a potential advantage over drug monotherapy in enhancing both the Total effective rate and PSQI score for insomnia. Among them, the integrated protocol of Tuina together with Breath guiding and acupoint application ranked highest in the network meta-analysis;however, given that the majority of evidence quality was assessed as low or very low, these findings are exploratory and further verification through high-quality RCTs is needed.

**Systematic review registration:**

https://www.crd.york.ac.uk/PROSPERO/view/CRD420261344347.

## 1 Introduction

Insomnia is defined as difficulty initiating sleep, difficulty maintaining sleep, or early morning awakening, occurring despite sufficient opportunity for sleep, and associated with marked daytime functional impairment or subjective distress (1). Globally, an estimated 16.2% of adults suffer from clinically significant insomnia symptoms (2), and sleep disturbances are also becoming increasingly prevalent in the general population (3). Insomnia not only compromises sleep quality but also markedly elevates the risk of multiple mental and somatic health disorders. Studies indicate that insomnia significantly raises the incidence of depression and anxiety, resulting in reduced psychological functioning and deficits in cognitive-emotional regulation (4). A meta-analysis reported that insomnia confers a 45% increase in the risk of cardiovascular events (5). Furthermore, several studies suggest that persistent insomnia symptoms are linked to a significantly higher risk of all-cause mortality (6). In addition, insomnia results in greater healthcare resource utilization and an increased economic burden (7). With the continuous increase in the population affected by sleep disorders, insomnia is attracting growing concern.

Existing treatments for insomnia are primarily categorized into pharmacotherapy (8) and cognitive-behavioral therapy (CBT) (9). Nevertheless, numerous studies have documented drug dependence, withdrawal reactions, and adverse effects including rebound insomnia and cognitive impairment (10–12). The use of hypnotic drugs is further accompanied by an elevated risk of death (13, 14). Although CBT is frequently recommended as a first-line alternative to pharmacotherapy, and its efficacy has been confirmed (15), its widespread adoption faces multiple obstacles, such as limited patient access time and a shortage of professionals trained in CBT, thereby hindering clinical implementation (16, 17). Hence, there is a clear need to identify safe and accessible alternative interventions.

While Tuina monotherapy is safe and non-invasive, its effectiveness may be restricted in patients with greater insomnia severity or prolonged disease course. Clinically, treatment does not rely solely on Tuina but frequently integrates it as a component of a multimodal approach.Despite a large body of clinical evidence on Tuina for insomnia, the majority of this evidence pertains to its use in combination with other modalities, and most studies consist of pairwise comparisons.The relative efficacy among different combined regimens remains unsubstantiated, thereby adding uncertainty to clinical decision-making. Which specific combinations of Tuina with other therapies provide the greatest effectiveness and safety remains unknown, posing a challenge in identifying the optimal therapeutic strategy.Consequently, to fill this key evidence gap, we performed a network meta-analysis to address the following clinical question: What are the comparative effectiveness and safety of Tuina-based combination therapies in improving outcomes for patients with insomnia?

## 2 Methods

### 2.1 Study design and registration

This study followed the PRISMA reporting guidelines for network meta-analyses (18), and its protocol was prospectively registered in PROSPERO (CRD420261344347).

### 2.2 Eligibility criteria

(1) Population: This study included adult participants who fulfilled the authoritative official diagnostic criteria for insomnia. To guarantee sample homogeneity, the following cases were systematically excluded: (i) secondary insomnia arising from pain, somatic disorders, psychiatric conditions, or environmental factors; (ii) insomnia presenting as an associated symptom of other diseases (e.g., sleep apnea syndrome, restless legs syndrome); and (iii) individuals with comorbid illnesses. No limitations were imposed regarding patients' sex, ethnic group, or length of disease course.

(2) Intervention: The experimental group was treated with Tuina in combination with other modalities (such as acupuncture, acupoint application, music, and others).

(3) Comparator: The control group was limited to pharmacotherapy, encompassing both benzodiazepine and non-benzodiazepine drugs.

(4) Outcomes: Primary outcomes: Total effective rate, calculated as [(number of cured cases+markedly effective cases+effective cases)/total number of cases] × 100%; Pittsburgh Sleep Quality Index (PSQI) (19). Secondary outcome: Safety, defined as [number of participants without adverse events/total number of cases] × 100%.

(5) Study Design:Randomized controlled trials (RCTs).

### 2.3 Search strategy

We conducted a systematic computerized search across PubMed, Cochrane, Embase, Web of Science, CNKI, VIP, WanFang, and SinoMed electronic databases, with the search timeframe extending until March 19, 2026. Two reviewers independently performed the literature search, and in the event of discrepancies, a third reviewer was consulted to reach a consensus. The full search strategy for each database is detailed in Supplementary Table 1. Furthermore, the reference lists of pertinent systematic reviews and meta-analyses were independently hand-searched to enhance the comprehensiveness of the retrieval.

### 2.4 Screening process and data extraction

All identified records were imported into EndNote 21.0 software, and duplicate publications were eliminated.Subsequently, two researchers, after verifying and removing duplicates, conducted a preliminary screening by reviewing titles and abstracts to exclude clearly unrelated articles. Thereafter, a secondary screening was performed by reading the full texts to decide on final inclusion. Data extraction was performed using a standardized Excel spreadsheet, capturing the following items:first author's name, year of publication, number of subjects, baseline characteristics of subjects, intervention/control regimens, treatment duration, outcome indicators, and outcome data. Two researchers independently extracted the data and conducted cross-verification. Any discrepancies were settled by discussion and negotiation, and the original articles were consulted as needed.

### 2.5 Quality assessment

The risk of bias and methodological quality of the included literature were evaluated using the ROB2 tool, as recommended in the Cochrane Handbook (Version 5.3.0) (20). ROB2 encompasses five domains of evaluation: bias in the randomization process, bias due to deviations from intended interventions, bias due to missing outcome data, bias in outcome measurement, and bias arising from selective outcome reporting. The risk of bias for each domain is categorized into three levels:low risk, unclear risk, and high risk. When all domains are determined to be at low risk, the overall risk of bias is low; when some domains show some risk and no domain is at high risk, the overall risk of bias is some risk; if any domain is judged as high risk, the overall risk of bias is high. Two independent reviewers conducted the quality evaluation, and any discrepancies were settled by consulting a third researcher to achieve consensus.

### 2.6 Statistical analysis

Stata version 16.0 was used for statistical analysis. For dichotomous outcomes, the relative risk (RR) was employed; for continuous outcomes, the mean difference (MD) served as the effect size, with 95% confidence intervals (CIs) computed. Network plots depicted the evidence network relations across interventions, with node size proportional to the total sample size of each treatment arm and line thickness indicating the number of studies providing direct comparisons between the corresponding interventions. Global consistency was evaluated using inconsistency models, and when local inconsistency was identified, the node-splitting method was employed for further analysis. The ranking of treatments was evaluated using the Surface Under the Cumulative Ranking Area (SUCRA), with higher values (ranging from 0% to 100%) reflecting a greater probability of being the best intervention. A league table was also produced to compare effect differences across interventions. Subgroup analysis and sensitivity analysis were performed to further explore the findings. Publication bias was evaluated via funnel plots, Begg's test, and Egger's test. Sensitivity analysis and publication bias testing were conducted solely for outcomes with more than 10 eligible studies.

### 2.7 Evaluation of evidence quality

The quality of evidence was evaluated using the CINeMA framework (21), which grades evidence across six domains: within-study bias, reporting bias, indirectness, imprecision, heterogeneity, and incoherence. The overall evidence quality was categorized into four grades: high, moderate, low, and very low.

## 3 Results

### 3.1 Study selection

**Figure 1:**
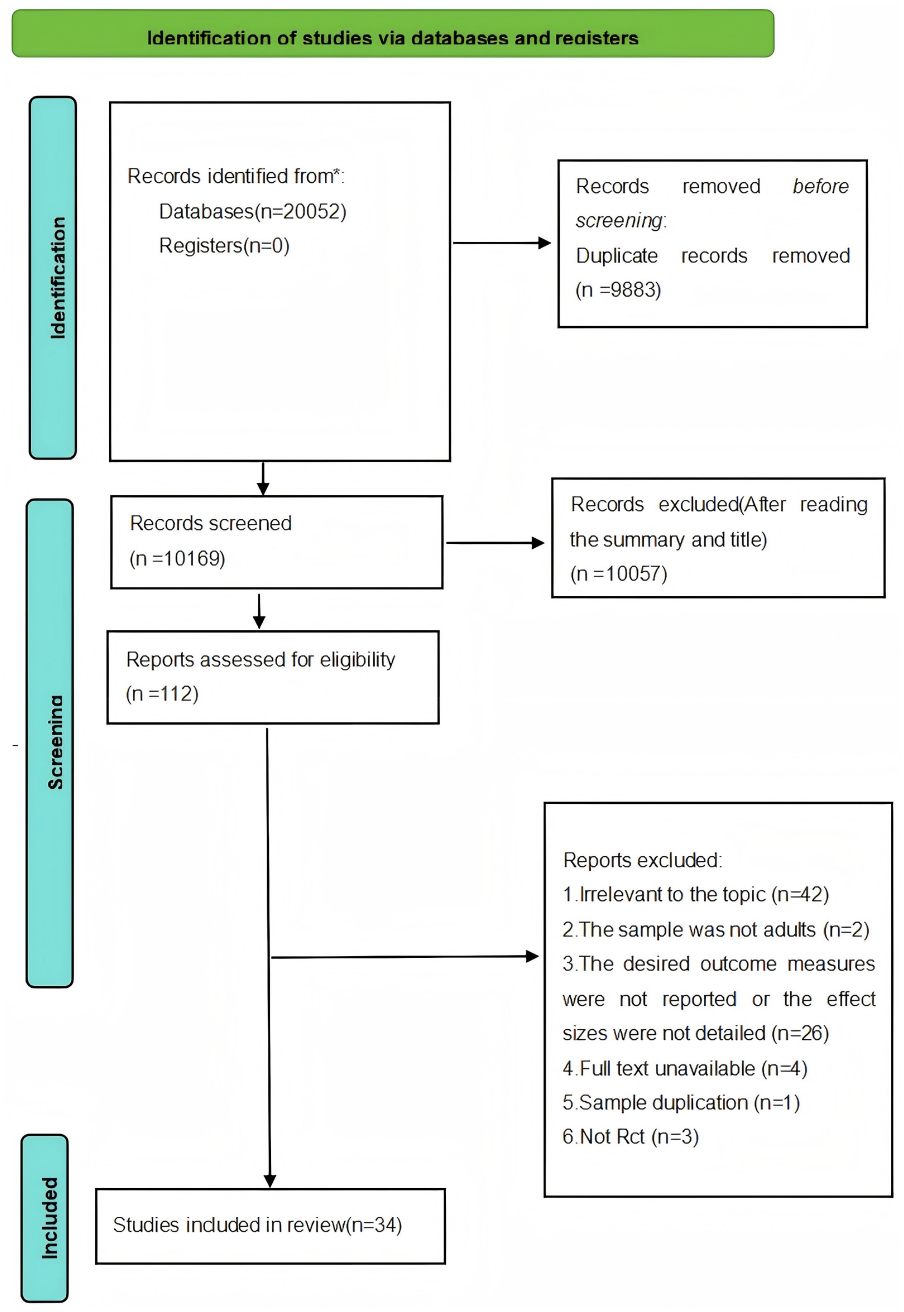
The PRISMA flowchart of the literature search and selection.

Figure 1 displays the flow diagram of the study screening process. The preliminary database search retrieved 20,052 records. At the title and abstract screening stage, 112 articles that potentially met the inclusion criteria were selected. Following additional full-text review and multi-step screening, 34 RCTs (22–55) satisfied the eligibility criteria and were incorporated into the final analysis.

### 3.2 Study characteristics

A total of 2,663 participants across 34 studies were enrolled in the included trials, and 12 interventions were evaluated: Tuina+acupuncture, Tuina+scraping, Tuina+breath guiding+acupoint application, Tuina+breath guiding, Tuina+music, Tuina+acupoint application, Tuina+acupoint application+scraping, Tuina+acupoint injection, Tuina+acupoint catgut embedding, Tuina+foot bath, Tuina+foot bath+acupoint application and Tuina+needle-embedding. The basic characteristics of the included studies are presented in Table 1.

**Table 1:** Characteristics of included studies.

Study ID	Sex (male/Female)		Average age (T/C)	Course of disease (Year) (T/C)	Sample size (T/C)	Intervention (T/C)	Course	Outcome
	T	C						
Huang et al. (22)	14/16	13/17	44.03 ± 9.33/44.30 ± 10.61	1.75 ± 1.78/1.83 ± 1.99	30/30	2 vs. 1	2 Weeks	
Chen and Liu (23)	33/27	31/29	52.32 ± 7.82/51.87 ± 7.71	—	60/60	2 vs. 1	2 Weeks	
Zhang and Li (24)	12/18	14/16	47.64 ± 5.78/45.19 ± 5.25	0.54 ± 0.11/0.57 ± 0.10	30/30	2 vs. 1	4 Weeks	
Wang et al. (25)	9/11	8/12	40.55 ± 14.78/40.07 ± 15.78	0.93 ± 0.39/1.02 ± 0.32	20/20	2 vs. 1	20 Days	
Gao et al. (26)	20/22	22/20	39.59 ± 5.05/39.54 ± 5.01	1.49 ± 0.13/1.47 ± 0.12	42/42	2 vs. 1	4 Weeks	
Ma et al. (27)	18/24	15/23	42 ± 16/43 ± 18	2.60 ± 2.39/2.47 ± 2.18	42/38	2 vs. 1	35 Days	
Gao (28)	32/22	30/24	43.03 ± 2.54/43.19 ± 2.63	3.59 ± 1.22/3.72 ± 1.38	54/54	2 vs. 1	30 Days	
Yang and Jin (29)	42/64	20/24	—	—	106/44	2 vs. 1	30 Days	
Wen and Chen (30)	17/23	19/21	—	—	40/40	2 vs. 1	30 Days	
Ruan et al. (31)	12/24	12/14	51.2/57.5	—	36/26	2 vs. 1	30 Days	
He et al. (32)	16/19	17/18	—	—	35/35	2 vs. 1	30 Days	
Jing (33)	20/26	16/29	—	—	46/45	2 vs. 1	36 Days	
Shen (34)	—	—	48.3 ± 9.71/47.167 ± 11.65	3.27 ± 3.06/3.19 ± 2.75	47/46	2 vs. 1	30 Days	
Ke (35)	10/13	8/15	46.2 ± 10.3/45.9 ± 11.4	7.5 ± 2.7/7.9 ± 2.1	23/23	2 vs. 1	20 Days	
Jiao et al. (36)	35/30	34/31	43.06 ± 3.22/42.36 ± 3.19	5.71 ± 0.80/5.58 ± 0.79	65/65	3 vs. 1	4 Weeks	
Wang et al. (37)	11/17, 12/18	13/17	44.86 ± 6.28, 44.17 ± 6.52/45.07 ± 5.47	2.46 ± 0.41, 2.40 ± 0.49/2.37 ± 0.42	28, 30/30	4, 5 vs. 1	4 Weeks	
Di et al. (38)	18/12	17/13	46.54 ± 5.78/46.47 ± 5.69	0.88 ± 0.21/0.87 ± 0.19	30/30	5 vs. 1	30 Days	
Fu et al. (39)	20/23	19/24	20.3 ± 2.1/20.2 ± 2.4	0.64 ± 0.43/0.62 ± 0.44	43/43	6 vs. 1	30 Days	
Chen et al. (40)	32/23	30/25	59.1 ± 9.5/57.5 ± 8.3	7.4 ± 2.2/7.2 ± 2.1	55/55	6 vs. 1	30 Days	
Hu (41)	10/20	13/17	—	—	30/30	7 vs. 1	20 Days	
Li et al. (42)	18/24	19/23	45.77 ± 8.69/46.35 ± 9.67	0.97 ± 0.21/0.86 ± 0.21	42/42	7 vs. 1	20 Days	
Zhou (43)	10/20	13/17	43.07 ± 9.28/42.53 ± 6.14	1.03 ± 0.46/1.05 ± 0.44	30/30	7 vs. 1	4 Weeks	
Liu (44)	20/30	21/29	—	—	50/50	7 vs. 1	3 Months	
Du (45)	22/18	19/21	43.67 ± 5.10/42.82 ± 5.40	1.67 ± 0.27/1.60 ± 0.34	40/40	7 vs. 1	2 Months	(22)
Chen (46)	16/24	17/23	39.56 ± 4.27/40.65 ± 4.13	—	40/40	8 vs. 1	4 Weeks	
Su et al. (47)	15/9	14/9	56.08 ± 8.04/55.96 ± 8.48	0.26 ± 0.12/0.33 ± 0.09	24/23	9 vs. 1	2 Weeks	
Huang and Huang (48)	7/18	7/14	45.0 ± 10.3/45.0 ± 10.5	4.52 ± 5.76/4.23 ± 3.82	25/24	10 vs. 1	30 Days	
Wei (49)	11/14	10/14	49.0 ± 10.4/47.0 ± 10.8	4.44 ± 5.77/4.31 ± 3.91	25/24	10 vs. 1	30 Days	
Yang and Zhan (50)	16/14	17/13	46.51 ± 11.73/45.73 ± 10.25	0.17 ± 0.1/0.16 ± 0.1	30/30	11 vs. 1	4 Weeks	
Liu (51)	15/15	17/13	50.14 ± 10.41/48.82 ± 11.3	11.35 ± 1.83/10.86 ± 2.24	30/30	11 vs. 1	30 Days	
Zhang (52)	16/24	17/23	40.3 ± 2.43/41 ± 3.13	—	40/40	11 vs. 1	20 Days	
Zhangand Liu (53)	—	—	—	—	40/30	11 vs. 1	1 Weeks	
Cheng (54)	32/44	22/28	47.2/46.8	4.5/4.7	76/50	12 vs. 1	4 Weeks	
Ye (55)	8/12	6/14	60.45 ± 8.35/59.80 ± 8.13	0.93 ± 0.49/0.88 ± 0.41	20/20	13 vs. 1	20 Days	

Outcome measures represent the following: Total Effective Rate; PSQI (Pittsburgh Sleep Quality Index); Safety. 1 = Drug therapy; 2 = Tuina+acupuncture; 3 = Tuina+scraping; 4 = Tuina+breath guiding+acupoint application; 5 = Tuina+breath guiding; 6 = Tuina+music; 7 = Tuina+acupoint application; 8 = Tuina+acupoint application+scraping; 9 = Tuina+acupoint injection; 10 = Tuina+acupoint catgut embedding; 11 = Tuina+foot bath; 12 = Tuina+foot bath+acupoint application; 13 = Tuina+needle-embedding.

### 3.3 Risk of bias in studies

**Figure 2:**
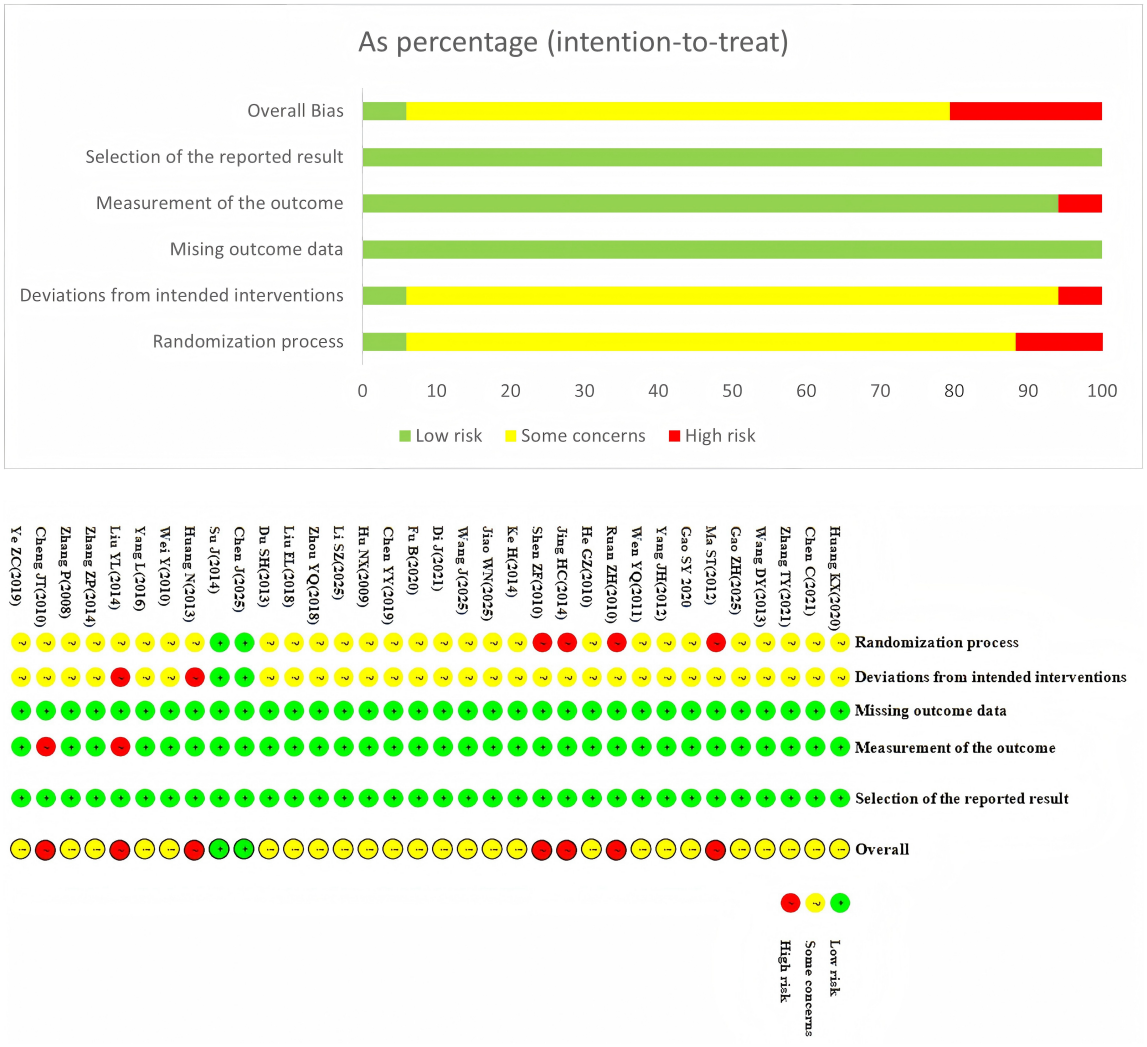
Summary results on the risk of bias (using RoB 2.0) of the included RCTs. Percent of studies with categories for risk of bias.

Among the included studies, 30 employed the random number table method, 1 used grouping according to visit sequence, and 3 did not report the specific randomization approach. Only 2 studies provided information on allocation concealment and the implementation of blinding. Outcome data were complete for all studies. The assessment of risk of bias revealed that the overall methodological quality of the included studies was at moderate risk. Specifically, 25 studies (73.5%) were judged as having moderate risk, 7 (20.6%) as high risk, and 2 (5.9%) as low risk. The risk of bias for each included study is displayed in Figure 2.

### 3.4 Network meta-analysis results

#### 3.4.1 Network plots

**Figure 3:**
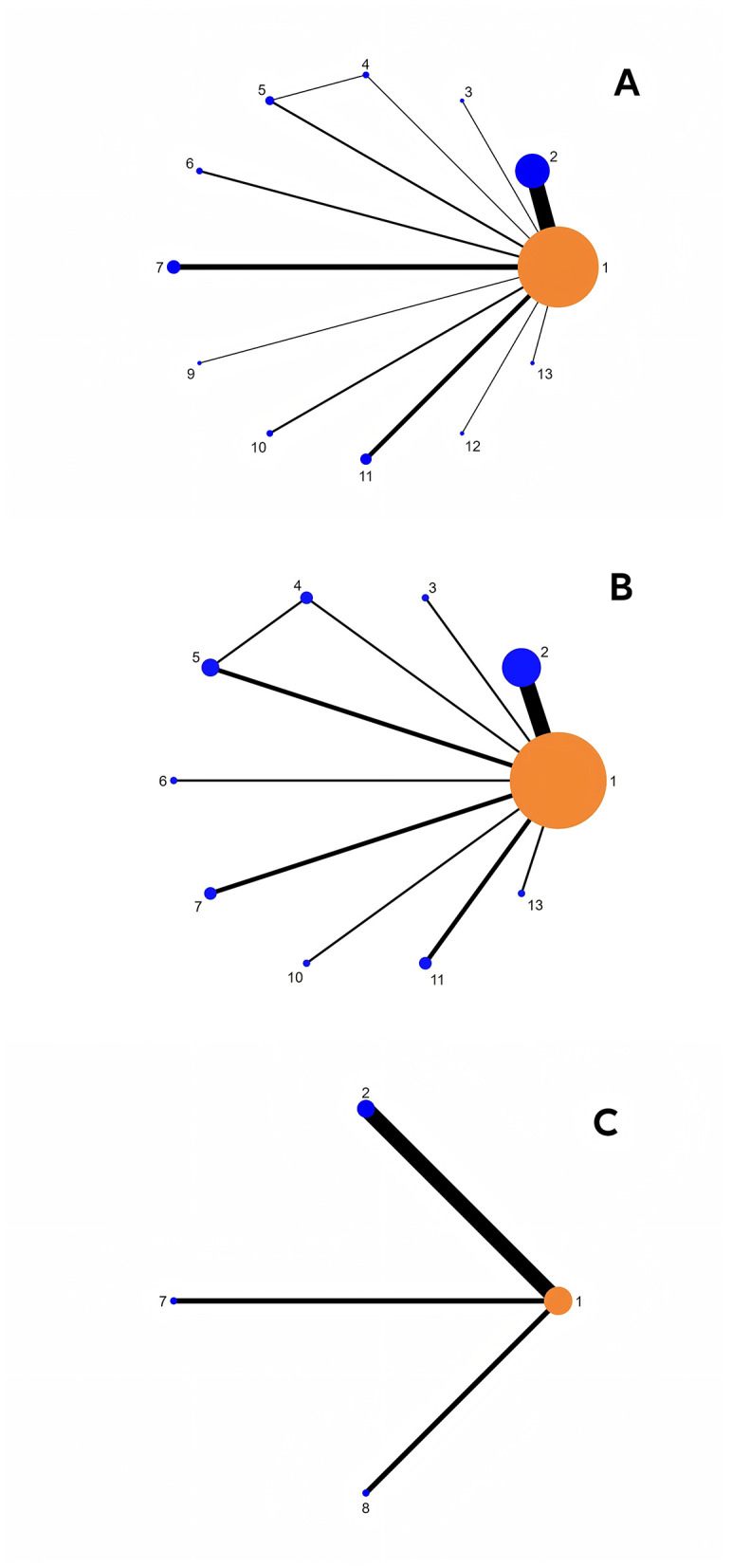
Network plots for Total Effective Rate **(A)**, PSQI **(B)**, safety **(C)**. 1 = Drug therapy; 2 = Tuina+acupuncture; 3 = Tuina+scraping; 4 = Tuina+breath guiding+acupoint application; 5 = Tuina+breath guiding; 6 = Tuina+music; 7 = Tuina+acupoint application; 8 = Tuina+acupoint application+scraping; 9 = Tuina+acupoint injection; 10 = Tuina+acupoint catgut embedding; 11 = Tuina+foot bath; 12 = Tuina+foot bath+acupoint application; 13 = Tuina+needle-embedding.

First, we examined the results for each outcome indicator via network plots. Figure 3 illustrates the network architecture of all competing interventions under each outcome measure. Each circle denotes a distinct intervention, where the node diameter is proportional to the total sample size of each intervention group, and the thickness of each line represents the number of studies making direct comparisons between the corresponding treatments.

#### 3.4.2 Total effective rate

**Figure 4:**
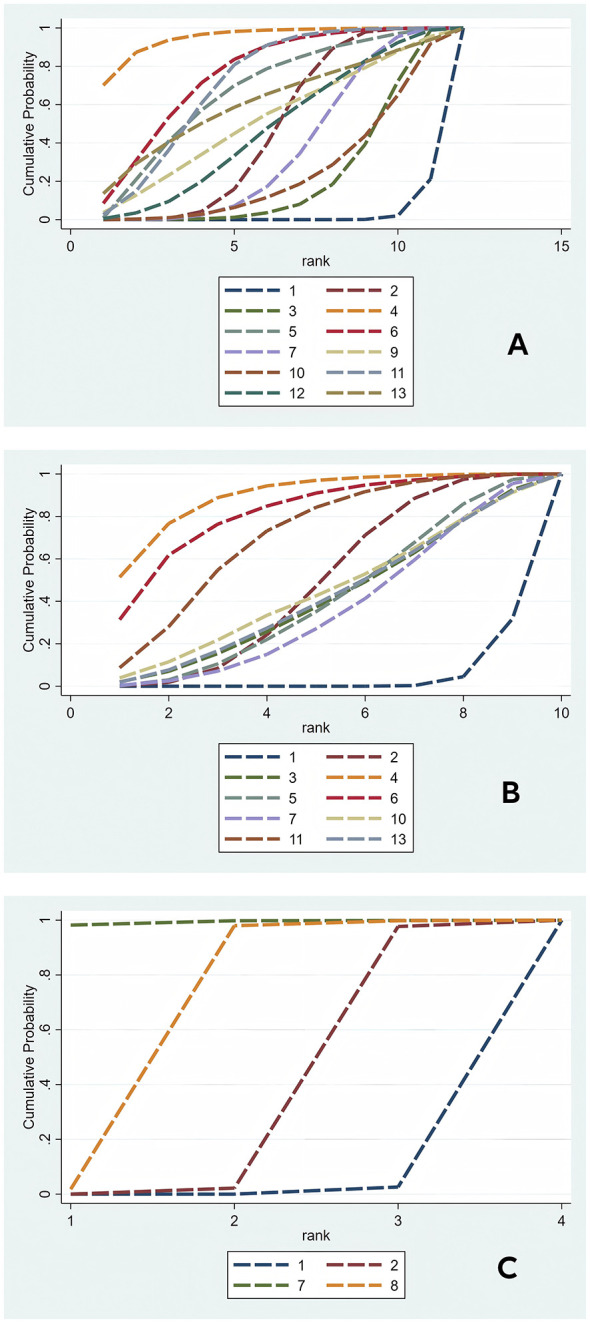
Net meta-analysis ranking results for each outcome indicator. Total Effective Rate **(A)**, PSQI **(B)**, safety **(C)**. 1 = Drug therapy; 2 = Tuina+acupuncture; 3 = Tuina+scraping; 4 = Tuina+breath guiding+acupoint application; 5 = Tuina+breath guiding; 6 = Tuina+music; 7 = Tuina+acupoint application; 8 = Tuina+acupoint application+scraping; 9 = Tuina+acupoint injection; 10 = Tuina+acupoint catgut embedding; 11 = Tuina+foot bath; 12 = Tuina+foot bath+acupoint application; 13 = Tuina+needle-embedding.

Among the 32 RCTs that reported Total Effective Rate, 11 distinct interventions were assessed, comprising 2,530 subjects. The network plots for these interventions is presented in Figure 3A. Initially, an inconsistency model was employed for testing, which yielded *P* > 0.05, suggesting no statistically significant global inconsistency. Consequently, the consistency model was adopted for subsequent analysis. Thereafter, local inconsistency was examined using the node-splitting method, and the results again showed *P* > 0.05, which suggests that no local inconsistency existed. The network meta-analysis demonstrated that the following interventions were significantly more effective than oral medication (*P* < 0.05): Tuina+breath guiding+acupoint application, Tuina+music, Tuina+foot bath, Tuina+breath guiding, Tuina+foot bath+acupoint application, Tuina+acupuncture, Tuina+acupoint application, and Tuina+scraping (Supplementary Table 2.1). Based on the SUCRA, the three highest-ranked interventions were Tuina+breath guiding+acupoint application (94.9%), Tuina+music (75.4%), and Tuina+foot bath (70.9%) (Figure 4A; Table 2).

**Table 2:** Ranking of efficacy of each treatments.

Treatment	Total effective rate		PSQI		Safety	
	SUCRA (%)	Rank	SUCRA (%)	Rank	SUCRA (%)	Rank
1	2.1	12	4.1	10	0.9	4
2	47.0	8	48.8	4	33.3	3
3	21.9	11	41.2	8	—	—
4	94.9	1	89.6	1	—	—
5	66.7	4	41.3	7	—	—
6	75.4	2	81.8	2	—	—
7	36.2	9	36.4	9	99.4	1
8	—	—	—	—	66.5	2
9	52.0	6	—	—	—	—
10	24.5	10	44.5	5	—	—
11	70.9	3	70.6	3	—	—
12	47.3	7	—	—	—	—
13	61.0	5	41.8	6	—	—

1 = Drug therapy; 2 = Tuina+acupuncture; 3 = Tuina+scraping; 4 = Tuina+breath guiding+acupoint application; 5 = Tuina+breath guiding; 6 = Tuina+music; 7 = Tuina+acupoint application; 8 = Tuina+acupoint application+scraping; 9 = Tuina+acupoint injection; 10 = Tuina+acupoint catgut embedding; 11 = Tuina+foot bath; 12 = Tuina+foot bath+acupoint application; 13 = Tuina+needle-embedding.

#### 3.4.3 PSQI

Among the 17 RCTs that reported PSQI, 9 distinct interventions were assessed, comprising 1,305 subjects. The network plots for these interventions is displayed in Figure 3B. Initially, an inconsistency model was employed for testing, yielding *P* > 0.05, which indicated no significant global inconsistency. Consequently, the consistency model was used for analysis. Thereafter, the node-splitting method was applied for local inconsistency testing, and the results again showed *P* > 0.05, which confirms that no local inconsistency existed. The network meta-analysis demonstrated that the following interventions were significantly more effective than oral medication (*P* < 0.05): Tuina+breath guiding+acupoint application, Tuina+music, Tuina+foot bath, and Tuina+acupuncture (Supplementary Table 2.2). Based on the SUCRA, the three highest-ranked interventions were Tuina+breath guiding+acupoint application (89.6%), Tuina+music (81.8%), and Tuina+foot bath (70.6%) (Figure 4B; Table 2).

#### 3.4.4 Safety

Five RCTs reported on safety, assessing 3 distinct interventions comprising 309 subjects. The network plots for these interventions is presented in Figure 3C. The inconsistency test yielded *P* > 0.05, and no closed loop was observed in the network plot; consequently, the consistency model was adopted for analysis. The network meta-analysis demonstrated that all interventions were safer than oral medication (*P* < 0.05) (Supplementary Table 2.3). Based on the SUCRA, the three highest-ranked interventions were Tuina+acupoint application (99.4%), Tuina+scraping+acupoint application (66.5%), and Tuina+acupuncture (33.3%) (Figure 4C; Table 2).

### 3.5 Publication bias analysis

**Figure 5:**
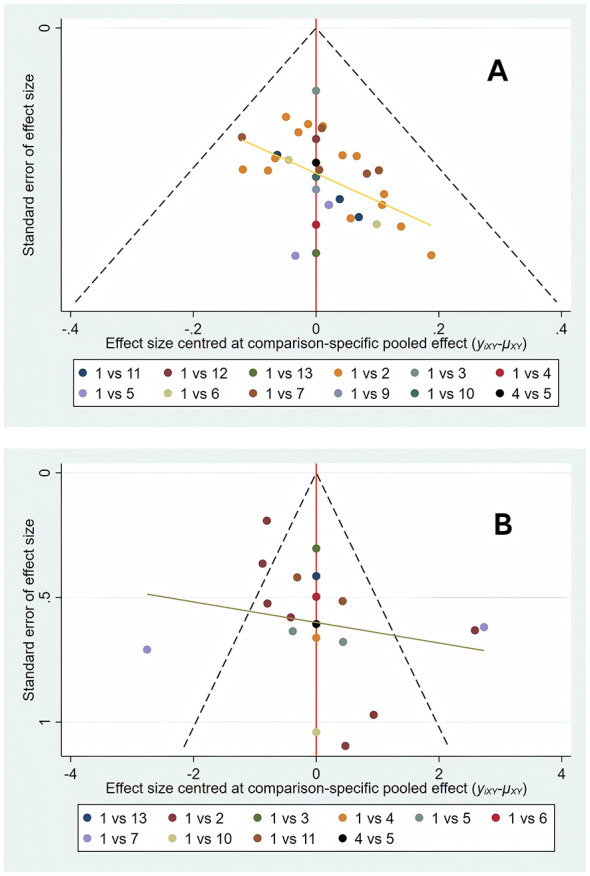
Comparison-correction funnel plot of each outcome indicator. Total Effective Rate **(A)**, PSQI **(B)**.

Publication bias was assessed for the total effective rate and PSQI outcomes. The findings indicated a high probability of publication bias for the total effective rate (*P* < 0.05), whereas the probability of publication bias for PSQI was low (*P* > 0.05) (Figure 5; Table 3).

**Table 3:** Results of the publication bias test.

Treatment	Total effective rate	PSQI
Egger	0.03	0.41
Begg-Mazumdar	0.07	0.52

### 3.6 Evaluation of evidence quality

Evidence quality was evaluated using the CINeMA framework. The findings indicated 6 of moderate quality, 4 of low quality, and 12 of very low quality (Supplementary Table 3). The main reasons for downgrading are within-study bias, imprecision and incoherence.

## 4 Discussion

### 4.1 Main finding

In total, 34 eligible RCTs were incorporated, assessing 12 distinct Tuina-based combined interventions. By means of network meta-analysis, we assessed three outcome measures—Total effective rate, PSQI score, and safety—to determine the best Tuina combination strategy on the basis of comparative effectiveness. The findings demonstrated that: (i) For Total effective rate, the three most effective strategies were Tuina+breath guiding+acupoint application (94.9%), Tuina+music (75.4%), and Tuina+foot bath (70.9%), each being significantly better than drug therapy. (ii) For PSQI score improvement, the same three interventions—Tuina+breath guiding+acupoint application (89.6%), Tuina+music (81.8%), and Tuina+foot bath (70.6%)—again occupied the top three positions, demonstrating strong concordance with the Total effective rate ranking. (iii) In an exploratory safety analysis limited to 5 studies and 3 interventions, the adverse event rates for Tuina+acupoint application (99.4%), Tuina+scraping+acupoint application (66.5%), and Tuina+acupuncture (33.3%) appeared lower than those for drugs. This sparse evidence network precludes any definitive comparative safety conclusions. Nevertheless, the evidence quality for the highest-ranked intervention was predominantly assessed as “low”; therefore, caution is warranted when interpreting these findings.

SUCRA integrates direct and indirect evidence, thereby enabling interventions to attain high rankings provided that the network estimates are consistent and precise, even if the number of direct-comparison RCTs for some interventions is limited. For instance, despite being examined in only a small number of original studies, Tuina+breath guiding+acupoint application established indirect comparison pathways with other network nodes via several shared comparators, thereby increasing the robustness of its relative effect estimates—a finding mirrored in its persistently high SUCRA score. Moreover, neither the inconsistency model nor the node-splitting method identified significant inconsistency, thereby diminishing the risk that small-sample studies might inflate the estimated effect magnitudes. The league table of pairwise comparisons indicated that the differences between the highest-ranked interventions may not be statistically significant. This implies that while these strategies represent some of the most effective choices for their respective outcomes, they are not statistically superior to every alternative. Consequently, the SUCRA rankings ought to be interpreted as a collection of promising intervention approaches rather than as a definitive hierarchical ordering of effectiveness.

### 4.2 Comparison with other studies

A network meta-analysis indicated that Tuina may be the most effective intervention for improving outcomes such as total effective rate and PSQI (56). Existing systematic reviews and meta-analyses have also confirmed that Tuina combined with acupuncture and other therapies is superior to medication alone or acupuncture alone in improving the total effective rate and PSQI score in patients with insomnia (57–59). Additionally, an umbrella review also supported the efficacy of Tuina for insomnia (60). These studies confirm the clinical value of Tuina in the treatment of insomnia. Notably, the present study simultaneously compared 12 Tuina combination interventions through network meta-analysis and ranked their efficacy using three primary evaluation parameters:total effective rate, PSQI score, and safety. This provides the first evidence of the relative efficacy and safety rankings among different Tuina combination regimens. By building upon the aforementioned published systematic reviews and meta-analyses, the present study addresses the limitation of prior research, which only compared individual combination regimens against drugs, and supplies novel evidence-based guidance for choosing the most appropriate Tuina-based combined therapy in clinical settings.

### 4.3 Explanation of results

Proteomics-based analyses have demonstrated that Tuina ameliorates insomnia symptoms through the regulation of key proteins in the hypothalamus of insomniac rats, including presenilin-1, creatine kinase, and the gamma-aminobutyric acid receptor subunit α-5. The involved mechanisms are mainly associated with signaling pathways such as neuroactive ligand-receptor interactions and hematopoietic cell lineage (61). Furthermore, Tuina upregulates the hypothalamic contents of brain-gut peptides including substance P, galanin, and β-endorphin, ameliorates the organization state of hypothalamic astrocytes, and modulates hypothalamic function, thus effectively improving sleep disturbances in insomnia model rats (62). In addition, Tuina decreases serum dopamine and acetylcholine concentrations, elevates 5-hydroxytryptamine (5-HT) levels, and corrects sleep-wake cycle dysregulation (63); it also lowers norepinephrine (NE) content and re-establishes the 5-HT/NE equilibrium (64). Furthermore, Tuina remodels the intestinal microbiota composition in insomniac rats, concurrently modulates brain-gut peptide levels across several brain regions and the colon, and attenuates hippocampal neuronal injury, thus substantially extending total sleep time (65). Collectively, these findings suggest that Tuina exerts its anti-insomnia effects via a multi-targeted integrated mechanism, including modulation of hypothalamic key proteins and brain-gut peptides, neurotransmitter balancing, gut microbiota remodeling, and attenuation of neuronal injury.

Breath guiding affects the sleep-wake cycle through the regulation of breathing patterns, with its mechanism involving effects on the brainstem respiratory center and sleep-modulating nuclei. In a murine model of urethane anesthesia, breathing frequency and variability show state-dependent characteristic alterations, suggesting a close neural coupling between respiratory rhythm and sleep-wake states (66). Slow and deep abdominal breathing decreases sympathetic activity and increases vagal tone. Extended inspiratory time markedly affects the autonomic regulation of the sinoatrial node, leading to changes in heart rate variability (67). Through activation of the parasympathetic nervous system, Breath guiding lowers plasma NE concentrations and diminishes oxidative stress biomarkers. This mechanism has been demonstrated to reduce sympathetic outflow in a study of exercise training in rats fed a high-fat diet (68). In a chronic intermittent hypoxia rat model, breathing disruptions that simulate sleep apnea activate the Hypothalamic-Pituitary-Adrenal axis and elevate the secretion of corticotropin-releasing hormone, adrenocorticotropic hormone, and corticosterone. Regular breathing training counteracts this stress response by maintaining blood gas homeostasis (69). Moreover, the respiratory pattern regulated by Breath guiding preserves the hypothalamic sleep-control center via enhancement of cerebral vascular endothelial function (70).

Acupoint application simultaneously upregulates 5-HT1A receptor mRNA expression and downregulates 5-HT2A receptor mRNA expression in the hypothalamus, brainstem, and hippocampus. Via this dual modulation of 5-HT1A and 5-HT2A receptors, it regulates the central serotonergic neurotransmitter system and improves sleep-wake cycle disruption; furthermore, transdermal permeation studies have demonstrated that its active ingredients penetrate the skin barrier, thus collectively providing both transdermal absorption and local acupoint stimulation (71). In addition, acupoint application markedly elevates Gamma-Aminobutyric Acid levels in the hypothalamus, brainstem, and hippocampus of insomnia model rats, thereby decreasing neuronal hyperexcitability (72).

In recent years, Tuina as a non-drug intervention has gained systematic endorsement from international bodies including the World Health Organization (WHO), and a growing number of countries have started to incorporate complementary medicine services such as Tuina into their medical insurance coverage (73, 74). The Global Traditional Medicine Strategy 2025–2034 (75)underscores the importance of advancing traditional, complementary, and integrative medicine within universal health coverage via evidence-based research, proper regulation, and health system integration, thus setting the course for the worldwide advancement of Tuina therapy for insomnia. Furthermore, as the WHO International Traditional Medicine Clinical Trial Registry Platform becomes more established, multi-center RCTs of Tuina for insomnia will benefit from global collaboration and data standardization, thereby significantly advancing the high-quality generation of traditional medicine evidence.

### 4.4 Limitations

Several limitations of this study should be noted: (i) All enrolled studies originated from China. Given potential differences in physical constitution, sleep practices, and receptiveness to Tuina between Chinese populations and other ethnic or cultural groups, the external validity of our results is unclear. (ii) While the SUCRA approach improves ranking stability through the use of indirect evidence, the majority of network nodes are supported by only a few small-sample studies, leading to broad confidence intervals around the effect estimates. Moreover, the restricted number of studies precluded prespecified subgroup or sensitivity analyses for the highest-ranked interventions, thereby diminishing the robustness of the findings. (iii) There was heterogeneity among studies regarding disease duration, age, intervention length, and Tuina operational parameters (force, frequency). (iv) The reporting of adverse events was generally inadequate and inconsistent across studies, with most failing to describe monitoring procedures or provide any safety data. This led to a sparse network for safety outcomes, preventing comparative safety assessments for the majority of interventions. The safety analysis was explicitly exploratory. Readers should not over-interpret the SUCRA-based safety rankings as definitive evidence of superiority. (v) The risk of bias evaluation indicated that the included RCTs exhibited varying degrees of shortcomings in random sequence generation, allocation concealment, and blinding procedures. Studies of lower methodological quality may compromise the stability of the findings.

## 5 Conclusion

Tuina-based combination therapies showed a potential advantage over drug monotherapy in enhancing both the Total effective rate and PSQI score for insomnia. Among them, the integrated protocol of Tuina together with Breath guiding and acupoint application ranked highest in the network meta-analysis. Nevertheless, constrained by the methodological quality, clinical heterogeneity, and relatively small sample sizes of the enrolled studies, these findings warrant confirmation through further high-quality, large-scale, multi-center RCTs.

## Data Availability

The original contributions presented in the study are included in the article/Supplementary material, further inquiries can be directed to the corresponding authors.
