# Supplementary material for: Tuina combined with other therapies for treating insomnia: a systematic review and network meta-analysis
Source: Front Neurol. 2026 May 28;17:1855450. doi: 10.3389/fneur.2026.1855450 (PMC13253231; doi:10.3389/fneur.2026.1855450)
Supplement: Supplementary file 1 [file Table_1.docx]

Supplementary Material

**Supplementary Table 1. Search strategy**

**Pubmed**

| **Search number** | **Query** |
| --- | --- |
| 1 | Massage OR Chinese manipulation OR tuina OR Tuina OR anmo OR manual therapy OR Manipulation OR rubbing abdomen OR abdominal massage OR Abdomen massage[Title/Abstract] |
| 2 | Sleep Initiation and Maintenance Disorders[MeSH Terms] |
| 3 | Sleep Initiation and Maintenance Disorders OR Insomnia OR Insomnia* OR sleep OR Sleep* OR Sleepless OR sleep disorder OR sleep problem OR Dyssomnias[Title/Abstract] |
| 4 | 2 OR 3 |
| 5 | 1 AND 4 |

**Web of science**

| **Search number** | **Query** |
| --- | --- |
| 1 | TS=(Massage OR Chinese manipulation OR tuina OR Tuina OR anmo OR manual therapy OR Manipulation OR rubbing abdomen OR abdominal massage OR Abdomen massage) |
| 2 | TS=(Sleep Initiation and Maintenance Disorders OR Insomnia OR Insomnia* OR sleep OR Sleep* OR Sleepless OR sleep disorder OR sleep problem OR Dyssomnias) |
| 3 | 1 AND 2 |

**cochrane library**

| **Search number** | **Query** |
| --- | --- |
| 1 | Massage OR Chinese manipulation OR tuina OR Tuina OR anmo OR manual therapy OR Manipulation OR rubbing abdomen OR abdominal massage OR Abdomen massage[Title/Abstract] |
| 2 | Sleep Initiation and Maintenance Disorders[MeSH Terms] |
| 3 | Sleep Initiation and Maintenance Disorders OR Insomnia OR Insomnia* OR sleep OR Sleep* OR Sleepless OR sleep disorder OR sleep problem OR Dyssomnias[Title/Abstract] |

**Embase**

| **Search number** | **Query** |
| --- | --- |
| 1 | Massage OR Chinese manipulation OR tuina OR Tuina OR anmo OR manual therapy OR Manipulation OR rubbing abdomen OR abdominal massage OR Abdomen massage[Title/Abstract] |
| 2 | Sleep Initiation and Maintenance Disorders[MeSH Terms] |
| 3 | Sleep Initiation and Maintenance Disorders OR Insomnia OR Insomnia* OR sleep OR Sleep* OR Sleepless OR sleep disorder OR sleep problem OR Dyssomnias[Title/Abstract] |
| 4 | 2 OR 3 |
| 5 | 1 AND 4 |

**CKNI**

TKA=('失眠'+'不寐'+'睡眠障碍'+'入睡困难'+'睡眠质量') AND TKA=('推拿'+'按摩 '+'手法'+'按跷'+'捏脊'+'摩腹'+'滚法'+'推法'+'揉法'+'摩法'+'擦法'+'搓法'+'抹法'+'按法'+'点法'+'捏法'+'拿法'+'捻法'+'拍法'+'击法'+'拨法'+'抖法'+'振法'+'指压')

**WanFang Data**

主题:(失眠 OR 不寐 OR 睡眠障碍 OR 入睡困难 OR 睡眠质量) AND 主题:(推拿 OR 按摩 OR 手法 OR 按跷 OR 捏脊 OR 摩腹 OR 滚法 OR 推法 OR 揉法 OR 摩法 OR 擦法 OR 搓法 OR 抹法 OR 按法 OR 点法 OR 捏法 OR 拿法 OR 捻法 OR 拍法 OR 击法 OR 拨法 OR 抖法 OR 振法 OR 指压)

**VIP**

(M=(失眠 OR 不寐 OR 睡眠障碍 OR 入睡困难 OR 睡眠质量) or K=(失眠 OR 不寐 OR 睡眠障碍 OR 入睡困难 OR 睡眠质量)) AND (M=(推拿 OR 按摩 OR 手法 OR 按跷 OR 捏脊 OR 摩腹 OR 滚法 OR 推法 OR 揉法 OR 摩法 OR 擦法 OR 搓法 OR 抹法 OR 按法 OR 点法 OR 捏法 OR 拿法 OR 捻法 OR 拍法 OR 击法 OR 拨法 OR 抖法 OR 振法 OR 指压) or K=(推拿 OR 按摩 OR 手法 OR 按跷 OR 捏脊 OR 摩腹 OR 滚法 OR 推法 OR 揉法 OR 摩法 OR 擦法 OR 搓法 OR 抹法 OR 按法 OR 点法 OR 捏法 OR 拿法 OR 捻法 OR 拍法 OR 击法 OR 拨法 OR 抖法 OR 振法 OR 指压))

**SinoMed**

(失眠 OR 不寐 OR 睡眠障碍 OR 入睡困难 OR 睡眠质量[Common fields]) AND (推拿 OR 按摩 OR 手法 OR 按跷 OR 捏脊 OR 摩腹 OR 滚法 OR 推法 OR 揉法 OR 摩法 OR 擦法 OR 搓法 OR 抹法 OR 按法 OR 点法 OR 捏法 OR 拿法 OR 捻法 OR 拍法 OR 击法 OR 拨法 OR 抖法 OR 振法 OR 指压[Common fields])

**Supplementary Table 2.League Table**

**2.1 Total Effective Rate**

| 4 | 6 | 11 | 5 | 13 | 9 | 12 | 2 | 7 | 10 | 3 | 1 |
| --- | --- | --- | --- | --- | --- | --- | --- | --- | --- | --- | --- |
| 4 | 0.86 (0.66,1.12) | 0.84 (0.66,1.07) | 0.84 (0.70,1.00) | 0.82 (0.56,1.22) | 0.79 (0.57,1.08) | 0.77 (0.59,1.01) | 0.77 (0.62,0.96) | 0.74 (0.59,0.94) | 0.71 (0.54,0.92) | 0.71 (0.56,0.89) | 0.64 (0.52,0.80) |
| 1.16 (0.89,1.52) | 6 | 0.97 (0.80,1.18) | 0.97 (0.76,1.26) | 0.96 (0.67,1.37) | 0.91 (0.69,1.21) | 0.89 (0.71,1.12) | 0.89 (0.76,1.05) | 0.87 (0.73,1.03) | 0.82 (0.66,1.02) | 0.82 (0.69,0.99) | 0.75 (0.64,0.87) |
| 1.19 (0.93,1.53) | 1.03 (0.85,1.25) | 11 | 1.00 (0.79,1.26) | 0.98 (0.70,1.39) | 0.94 (0.72,1.21) | 0.92 (0.75,1.12) | 0.92 (0.81,1.04) | 0.89 (0.77,1.02) | 0.85 (0.70,1.02) | 0.85 (0.73,0.98) | 0.77 (0.68,0.86) |
| 1.19 (1.00,1.42) | 1.03 (0.80,1.32) | 1.00 (0.79,1.26) | 5 | 0.98 (0.67,1.44) | 0.94 (0.69,1.27) | 0.92 (0.71,1.18) | 0.92 (0.75,1.13) | 0.89 (0.72,1.10) | 0.85 (0.66,1.09) | 0.84 (0.68,1.05) | 0.77 (0.63,0.93) |
| 1.21 (0.82,1.79) | 1.04 (0.73,1.49) | 1.02 (0.72,1.43) | 1.02 (0.70,1.48) | 13 | 0.95 (0.64,1.42) | 0.93 (0.65,1.33) | 0.93 (0.67,1.29) | 0.90 (0.65,1.26) | 0.86 (0.60,1.23) | 0.86 (0.61,1.20) | 0.78 (0.56,1.07) |
| 1.27 (0.93,1.75) | 1.09 (0.83,1.45) | 1.07 (0.82,1.38) | 1.07 (0.79,1.45) | 1.05 (0.71,1.56) | 9 | 0.98 (0.74,1.29) | 0.98 (0.77,1.24) | 0.95 (0.74,1.21) | 0.90 (0.68,1.19) | 0.90 (0.70,1.15) | 0.82 (0.65,1.03) |
| 1.30 (0.99,1.70) | 1.12 (0.90,1.40) | 1.09 (0.90,1.33) | 1.09 (0.85,1.41) | 1.07 (0.75,1.54) | 1.02 (0.77,1.35) | 12 | 1.00 (0.85,1.18) | 0.97 (0.81,1.16) | 0.92 (0.74,1.15) | 0.92 (0.77,1.11) | 0.83 (0.71,0.98) |
| 1.30 (1.04,1.63) | 1.12 (0.95,1.32) | 1.09 (0.96,1.24) | 1.09 (0.89,1.34) | 1.07 (0.78,1.49) | 1.02 (0.81,1.30) | 1.00 (0.85,1.18) | 2 | 0.97 (0.88,1.06) | 0.92 (0.79,1.08) | 0.92 (0.83,1.02) | 0.84 (0.80,0.88) |
| 1.34 (1.07,1.69) | 1.16 (0.97,1.38) | 1.13 (0.98,1.29) | 1.13 (0.91,1.40) | 1.11 (0.80,1.54) | 1.06 (0.83,1.35) | 1.03 (0.86,1.23) | 1.03 (0.94,1.13) | 7 | 0.95 (0.80,1.13) | 0.95 (0.84,1.07) | 0.86 (0.80,0.93) |
| 1.41 (1.08,1.84) | 1.21 (0.98,1.51) | 1.18 (0.98,1.43) | 1.18 (0.92,1.52) | 1.16 (0.82,1.66) | 1.11 (0.84,1.46) | 1.08 (0.87,1.35) | 1.08 (0.93,1.27) | 1.05 (0.89,1.25) | 10 | 1.00 (0.84,1.19) | 0.91 (0.78,1.05) |
| 1.41 (1.12,1.79) | 1.21 (1.01,1.46) | 1.18 (1.02,1.37) | 1.18 (0.95,1.47) | 1.17 (0.83,1.63) | 1.11 (0.87,1.42) | 1.09 (0.90,1.30) | 1.09 (0.98,1.20) | 1.05 (0.93,1.19) | 1.00 (0.84,1.19) | 3 | 0.91 (0.83,0.99) |
| 1.56 (1.26,1.94) | 1.34 (1.15,1.57) | 1.31 (1.16,1.46) | 1.31 (1.07,1.60) | 1.29 (0.93,1.77) | 1.22 (0.97,1.54) | 1.20 (1.02,1.40) | 1.20 (1.14,1.26) | 1.16 (1.07,1.26) | 1.10 (0.95,1.28) | 1.10 (1.01,1.21) | 1 |

Note:1=Drug therapy; 2=Tuina+Acupuncture; 3=Tuina+Scraping; 4=Tuina+Breath guiding+Acupoint application; 5=Tuina+Breath guiding; 6=Tuina+Music; 7=Tuina+Acupoint application; 8=Tuina+Acupoint application+Scraping; 9=Tuina+Acupoint injection; 10=Tuina+Acupoint catgut embedding; 11=Tuina+Foot bath; 12=Tuina+Foot bath+Acupoint application；13=Tuina+needle-embedding.

**2.1 PSQI**

| 4 | 6 | 11 | 2 | 10 | 13 | 5 | 3 | 7 | 1 |
| --- | --- | --- | --- | --- | --- | --- | --- | --- | --- |
| 4 | 0.59 (-3.86,5.04) | 1.59 (-2.24,5.41) | 2.76 (-0.57,6.10) | 3.04 (-1.76,7.84) | 3.22 (-1.20,7.64) | 3.14 (0.07,6.21) | 3.22 (-1.16,7.61) | 3.42 (-0.46,7.31) | 5.34 (2.25,8.43) |
| -0.59 (-5.04,3.86) | 6 | 0.99 (-2.93,4.91) | 2.17 (-1.27,5.61) | 2.45 (-2.43,7.33) | 2.63 (-1.87,7.13) | 2.55 (-1.43,6.52) | 2.63 (-1.84,7.10) | 2.83 (-1.14,6.81) | 4.75 (1.54,7.96) |
| -1.59 (-5.41,2.24) | -0.99 (-4.91,2.93) | 11 | 1.18 (-1.40,3.76) | 1.46 (-2.85,5.77) | 1.64 (-2.25,5.52) | 1.55 (-1.70,4.81) | 1.64 (-2.21,5.48) | 1.84 (-1.42,5.10) | 3.76 (1.50,6.01) |
| -2.76 (-6.10,0.57) | -2.17 (-5.61,1.27) | -1.18 (-3.76,1.40) | 2 | 0.28 (-3.60,4.16) | 0.46 (-2.94,3.86) | 0.38 (-2.28,3.03) | 0.46 (-2.90,3.81) | 0.66 (-2.00,3.33) | 2.58 (1.33,3.83) |
| -3.04 (-7.84,1.76) | -2.45 (-7.33,2.43) | -1.46 (-5.77,2.85) | -0.28 (-4.16,3.60) | 10 | 0.18 (-4.67,5.03) | 0.10 (-4.26,4.45) | 0.18 (-4.63,4.99) | 0.38 (-3.98,4.74) | 2.30 (-1.37,5.97) |
| -3.22 (-7.64,1.20) | -2.63 (-7.13,1.87) | -1.64 (-5.52,2.25) | -0.46 (-3.86,2.94) | -0.18 (-5.03,4.67) | 13 | -0.08 (-4.02,3.85) | -0.00 (-4.44,4.44) | 0.20 (-3.74,4.14) | 2.12 (-1.04,5.28) |
| -3.14 (-6.21,-0.07) | -2.55 (-6.52,1.43) | -1.55 (-4.81,1.70) | -0.38 (-3.03,2.28) | -0.10 (-4.45,4.26) | 0.08 (-3.85,4.02) | 5 | 0.08 (-3.81,3.98) | 0.29 (-3.03,3.60) | 2.20 (-0.14,4.55) |
| -3.22 (-7.61,1.16) | -2.63 (-7.10,1.84) | -1.64 (-5.48,2.21) | -0.46 (-3.81,2.90) | -0.18 (-4.99,4.63) | 0.00 (-4.44,4.44) | -0.08 (-3.98,3.81) | 3 | 0.20 (-3.70,4.10) | 2.12 (-0.99,5.23) |
| -3.42 (-7.31,0.46) | -2.83 (-6.81,1.14) | -1.84 (-5.10,1.42) | -0.66 (-3.33,2.00) | -0.38 (-4.74,3.98) | -0.20 (-4.14,3.74) | -0.29 (-3.60,3.03) | -0.20 (-4.10,3.70) | 7 | 1.92 (-0.43,4.27) |
| -5.34 (-8.43,-2.25) | -4.75 (-7.96,-1.54) | -3.76 (-6.01,-1.50) | -2.58 (-3.83,-1.33) | -2.30 (-5.97,1.37) | -2.12 (-5.28,1.04) | -2.20 (-4.55,0.14) | -2.12 (-5.23,0.99) | -1.92 (-4.27,0.43) | 1 |

Note:1=Drug therapy; 2=Tuina+Acupuncture; 3=Tuina+Scraping; 4=Tuina+Breath guiding+Acupoint application; 5=Tuina+Breath guiding; 6=Tuina+Music; 7=Tuina+Acupoint application; 8=Tuina+Acupoint application+Scraping; 9=Tuina+Acupoint injection; 10=Tuina+Acupoint catgut embedding; 11=Tuina+Foot bath; 12=Tuina+Foot bath+Acupoint application；13=Tuina+needle-embedding.

**3.3 Safety**

| 7 | 8 | 2 | 1 |
| --- | --- | --- | --- |
| 7 | 0.60 (0.36,0.98) | 0.48 (0.30,0.76) | 0.44 (0.28,0.70) |
| 1.67 (1.02,2.75) | 8 | 0.80 (0.65,1.00) | 0.74 (0.61,0.91) |
| 2.08 (1.31,3.30) | 1.24 (1.00,1.54) | 2 | 0.92 (0.85,1.00) |
| 2.25 (1.43,3.54) | 1.34 (1.10,1.64) | 1.08 (1.00,1.17) | 1 |

Note:1=Drug therapy; 2=Tuina+Acupuncture; 3=Tuina+Scraping; 4=Tuina+Breath guiding+Acupoint application; 5=Tuina+Breath guiding; 6=Tuina+Music; 7=Tuina+Acupoint application; 8=Tuina+Acupoint application+Scraping; 9=Tuina+Acupoint injection; 10=Tuina+Acupoint catgut embedding; 11=Tuina+Foot bath; 12=Tuina+Foot bath+Acupoint application；13=Tuina+needle-embedding.

**Supplementary Table 3.CINeMA results**

**3.1 Total effective rate**

| **Comparison** | **Number of studies** | **Within-study bias** | **Reporting bias** | **Indirectness** | **Imprecision** | **Heterogeneity** | **Incoherence** | **Confidence rating** | **Reason(s) for downgrading** |
| --- | --- | --- | --- | --- | --- | --- | --- | --- | --- |
| 1:2 | 13 | Some concerns | Low risk | No concerns | No concerns | No concerns | No concerns | Moderate | ["Within-study bias"] |
| 1:3 | 1 | Some concerns | Low risk | No concerns | No concerns | No concerns | No concerns | Moderate | ["Within-study bias"] |
| 1:4 | 1 | Some concerns | Low risk | Some concerns | No concerns | No concerns | No concerns | Low | ["Within-study bias","Indirectness"] |
| 1:5 | 2 | Some concerns | Low risk | No concerns | No concerns | No concerns | No concerns | Moderate | ["Within-study bias"] |
| 1:6 | 2 | Some concerns | Low risk | No concerns | No concerns | No concerns | No concerns | Moderate | ["Within-study bias"] |
| 1:7 | 5 | Some concerns | Low risk | No concerns | No concerns | No concerns | No concerns | Moderate | ["Within-study bias"] |
| 1:9 | 1 | No concerns | Low risk | No concerns | Major concerns | No concerns | No concerns | Low | ["Imprecision"] |
| 1:10 | 2 | Major concerns | Low risk | No concerns | Major concerns | No concerns | No concerns | Very low | ["Within-study bias","Imprecision"] |
| 1:11 | 4 | Some concerns | Low risk | No concerns | No concerns | No concerns | No concerns | Moderate | ["Within-study bias"] |
| 1:12 | 1 | Major concerns | Low risk | No concerns | No concerns | No concerns | No concerns | Low | ["Within-study bias"] |
| 1:13 | 1 | Some concerns | Low risk | No concerns | Major concerns | No concerns | No concerns | Very low | ["Within-study bias","Imprecision"] |
| 4:5 | 1 | Some concerns | Low risk | Some concerns | Major concerns | No concerns | No concerns | Very low | ["Within-study bias","Indirectness","Imprecision"] |

Note:1=Drug therapy; 2=Tuina+Acupuncture; 3=Tuina+Scraping; 4=Tuina+Breath guiding+Acupoint application; 5=Tuina+Breath guiding; 6=Tuina+Music; 7=Tuina+Acupoint application; 8=Tuina+Acupoint application+Scraping; 9=Tuina+Acupoint injection; 10=Tuina+Acupoint catgut embedding; 11=Tuina+Foot bath; 12=Tuina+Foot bath+Acupoint application；13=Tuina+needle-embedding.

**3.2 PSQI**

| **Comparison** | **Number of studies** | **Within-study bias** | **Reporting bias** | **Indirectness** | **Imprecision** | **Heterogeneity** | **Incoherence** | **Confidence rating** | **Reason(s) for downgrading** |
| --- | --- | --- | --- | --- | --- | --- | --- | --- | --- |
| 1:2 | 7 | Some concerns | Low risk | No concerns | No concerns | Major concerns | Major concerns | Very low | ["Within-study bias","Heterogeneity","Incoherence"] |
| 1:3 | 1 | Some concerns | Low risk | No concerns | Major concerns | No concerns | Major concerns | Very low | ["Within-study bias","Imprecision","Incoherence"] |
| 1:4 | 1 | Some concerns | Low risk | Some concerns | No concerns | No concerns | No concerns | Low | ["Within-study bias","Indirectness"] |
| 1:5 | 2 | Some concerns | Low risk | Some concerns | No concerns | Major concerns | Major concerns | Very low | ["Within-study bias","Indirectness","Heterogeneity","Incoherence"] |
| 1:6 | 1 | Some concerns | Low risk | No concerns | No concerns | No concerns | Major concerns | Very low | ["Within-study bias","Incoherence"] |
| 1:7 | 2 | Some concerns | Low risk | No concerns | Major concerns | No concerns | Major concerns | Very low | ["Within-study bias","Imprecision","Incoherence"] |
| 1:10 | 1 | Some concerns | Low risk | No concerns | Major concerns | No concerns | Major concerns | Very low | ["Within-study bias","Imprecision","Incoherence"] |
| 1:11 | 2 | Some concerns | Low risk | No concerns | No concerns | No concerns | Major concerns | Very low | ["Within-study bias","Incoherence"] |
| 1:13 | 1 | Some concerns | Low risk | No concerns | Major concerns | No concerns | Major concerns | Very low | ["Within-study bias","Imprecision","Incoherence"] |
| 4:5 | 1 | Some concerns | Low risk | Some concerns | No concerns | Major concerns | No concerns | Very low | ["Within-study bias","Indirectness","Heterogeneity"] |

Note:1=Drug therapy; 2=Tuina+Acupuncture; 3=Tuina+Scraping; 4=Tuina+Breath guiding+Acupoint application; 5=Tuina+Breath guiding; 6=Tuina+Music; 7=Tuina+Acupoint application; 8=Tuina+Acupoint application+Scraping; 9=Tuina+Acupoint injection; 10=Tuina+Acupoint catgut embedding; 11=Tuina+Foot bath; 12=Tuina+Foot bath+Acupoint application；13=Tuina+needle-embedding.
